# Exploring the feasibility of evaluating a community alliance welfare advice programme co-located in primary care in Bradford: an uncontrolled before and after study

**DOI:** 10.1186/s12889-024-17773-x

**Published:** 2024-01-25

**Authors:** Sian Reece, Rachael H. Moss, Zahrah Tanveer, Mohammed Hammad, Kate E. Pickett, Josie Dickerson

**Affiliations:** 1 Hull York Medical School, York, North Yorkshire UK; 2 Bradford Institute for Health Research, Bradford Royal Infirmary, Bradford, West Yorkshire UK; 3 Department of Health Sciences, University of York, Heslington Road, York, North Yorkshire UK

**Keywords:** Co-location, Welfare advice, Primary care, General practice, Financial insecurity, Poverty, Mental health, Health inequalities, Social inequalities, Ethnicity

## Abstract

**Background:**

Welfare advice services co-located in health settings are known to improve financial security. However, little is known on how to effectively evaluate these services. This study aims to explore the feasibility of evaluating a welfare advice service co-located in a primary care setting in a deprived and ethnically diverse population. It seeks to investigate whether the proposed evaluation tools and processes are acceptable and feasible to implement and whether they are able to detect any evidence of promise for this intervention on the mental health, wellbeing and financial security of participants.

**Methods:**

An uncontrolled before and after study design was utilised. Data on mental health, wellbeing, quality of life and financial outcomes were collected at baseline prior to receiving welfare advice and at three months follow-up. Multiple logistic and linear regression models were used to explore individual differences in self-reported financial security and changes to mental health, wellbeing and quality of life scores before and after the provision of welfare advice.

**Results:**

Overall, the majority of key outcome measures were well completed, indicating participant acceptability of the mental health, wellbeing, quality of life and financial outcome measures used in this population. There was evidence suggestive of an improvement in participant financial security and evidence of promise for improvements in measured wellbeing and health-related quality of life for participants accessing services in a highly ethnically diverse population. Overall, the VCS Alliance welfare advice programme generated a total of £21,823.05 for all participants, with participants gaining an average of £389.70 per participant for participants with complete financial outcome data.

**Conclusions:**

This research demonstrates the feasibility of evaluating a welfare advice service co-located in primary care in a deprived and ethnically diverse setting utilising the ascribed mental health, wellbeing and quality of life and financial outcome tools. It provides evidence of promise to support the hypothesis that the implementation of a welfare advice service co-located in a health setting can improve health and wellbeing and reduce health inequalities.

## Background

The relationship between financial insecurity and poor health and wellbeing is well established. Early childhood deprivation is associated with poor physical and mental health and negative social outcomes that not only limit a child’s development in the short-term but have long lasting effects into adulthood [1]. In adulthood, links between financial difficulties, social deprivation and mental health are also well established [2]. Financial insecurity can precipitate and perpetuate mental health problems [2, 3] and has been found to be a predictor of chronic physical illness [4–6]. Furthermore, individuals suffering with poor mental health associated with financial insecurity, worsened in recent years by austerity and in the recovery from the global COVID-19 pandemic, are more likely to face challenges in accessing the advice and support needed to address such welfare issues [3, 6]

The adverse effect of financial insecurity on physical and mental health can be obviated if corrected early on [7]. Consequently, the availability and accessibility of welfare advice to improve uptake of the benefits and financial support to which individuals are eligible is crucial to addressing these social determinants of health and to improving health equity [5].

There is emerging evidence to suggest that there may be unequal access to, and uptake of, benefits and income support for those who are eligible and this has been found to be particularly pronounced in some ethnic minority groups [8–10]. There is also evidence from self-reported data that young families in the lowest income group claim fewer benefits than those in higher income bands but the reasons for this pattern are unclear [11]. Reasons for reduced uptake of welfare advice and financial support for vulnerable groups have included institutional discrimination, language and communication barriers and stigma [8–10]. There is a need for further research, providing empirical evidence to demonstrate the variation in uptake of welfare advice compared with eligibility, across the range of benefits and other financial support available and by key sociodemographics, most notably ethnic group. Further research is also needed on how best to improve the design and delivery of these services to improve access for the most vulnerable.

Integration of welfare advice services can help to ensure timely and targeted access in a time and place of need. Various schemes have been put in place to improve the accessibility and uptake of welfare advice and the receipt of benefits and other financial support by co-locating welfare advice services within health settings [12–14]. Welfare advice services co-located in health settings are collaborations between organisations specialising in welfare advice and health services. They offer potential benefits for both healthcare professionals and welfare advisors, in addition to the provision of welfare advice. Patients frequently present to healthcare professionals with social welfare problems, which may result from their health condition or are contributing to their illness [15]. Partnerships with welfare advice services can help healthcare professionals to address the social welfare needs of patients, which are beyond their expertise to manage [16]. For welfare advisors, partnerships with healthcare could facilitate intervention at an earlier stage, before social welfare problems escalate and can enable access to the medical information needed to support welfare casework and to advocate for systemic change [17–19]. On an individual level, patients are able to access welfare advice through the health service they are attending, benefitting from a co-ordinated and holistic response to their needs [20].

A systematic review, published in 2006, of welfare advice delivered in health settings found that there was evidence that this approach resulted in financial gains but at that time there was limited high quality evidence to determine whether this resulted in improved uptake of welfare advice or measurable health and social benefits [21]. Furthermore, none of the included studies considered variation in uptake or outcomes measures between ethnic groups. Since this time, a further narrative systematic review has been published, building upon the previously published systematic review and theory of change model [22]. This review demonstrated health and wellbeing, and improved financial security for participants. Overall, included studies reported a social return of investment of £27 per £1 invested. Improvements to health and wellbeing were attributed to action on key social determinants of health. Several challenges were highlighted relevant for future evaluations of co-located welfare advice services. Low statistical power was a common problem owing to low recruitment and retention of participants. Furthermore, the review highlights difficulties in the choice of suitable effectiveness and implementation outcome measures, resulting in significant heterogeneity in reported outcomes across evaluations of co-located welfare advice services. More recently published studies have also highlighted the challenge inherent in choice of appropriate follow-up time. Outcome measures collected at 24 months suggested where improvements might exist, they may not persist beyond this time [23]. The challenge of recruiting minority groups was also raised as a particular concern in many studies. Furthermore, given the overall, generally poor scientific quality of the studies, care must be taken in drawing firm conclusions about the impact of co-located services on health, social and financial outcomes from both systematic reviews of the existence evidence base in this area.

The Bradford City Clinical Commissioning Group commissioned a welfare advice programme co-located in the primary care network across Bradford as part of their Reducing Inequalities in Communities programme [24]. The welfare advice programme was co-ordinated by the Voluntary and Community Sector (VCS) Alliance and consisted of nine discrete welfare advice providers [25]. Six providers delivered general welfare advice services and three provided specialist welfare advice services. Specialist services were provided were provided for individuals and their families affected by cancer, disabled people and their families and the elderly population. Services were access through referral by a general practitioner.

The aim of this study is to explore the feasibility of evaluating a welfare advice service co-located in a health setting in general practice. It seeks to investigate whether the proposed evaluation tools and processes are acceptable and feasible to implement and where permitting, whether they are able to detect any evidence of promise for this intervention on the health, wellbeing and financial security of participants in an ethnically diverse and deprived population.

The objectives of this study are to:

- Explore the feasibility of recruiting and retaining participants for an evaluation of a welfare advice service co-located in a health setting within an ethnically diverse and deprived population.
- Explore the acceptability and utility of the proposed evaluation tools to evaluate the impact of this intervention on the health, wellbeing and financial security of participants with respect to completeness of outcome measures and their ability to detect change in outcome measures for the intervention in this population.
- Where the above outcomes permit, to explore the magnitude and direction of effect of the impact of the intervention on the health, wellbeing and financial security of participants that may also inform sample size calculations in future evaluations.

## Methods

### Study design

An uncontrolled before and after study design was utilised to conduct this evaluation. Data were collected by welfare advisors from participants at two time points: at baseline prior to receiving welfare advice at their first appointment with the welfare advisor; and at three months following their first appointment with the welfare advisor.

### Data collection

All individuals aged 18 + years who accessed the welfare advice service through referral by their GP during the recruitment period were eligible. Where a participant spoke a language other than English, written information was provided in additional commonly spoken languages as required, including Arabic, Bangla, Hebrew, Latvian, Malay, Polish, Romanian, Slovenian and Urdu. Participants were offered a £15 Love2Shop voucher [26] upon completion of their 3 month follow-up survey as a token of appreciation.

At their first appointment with the welfare advisor and prior to the provision of any welfare advice, all eligible clients were approached by their welfare advisor to seek consent for participation in the evaluation. After obtaining written consent, participants were asked to complete a baseline survey to assess their current levels of self-reported financial security and health and wellbeing prior to receiving their welfare advice. The surveys were self-reported and took approximately 10 min to complete.

Three months following their initial appointment with the welfare advisor, participants were asked to complete the same survey again. This usually occurred at their final appointment with their welfare advisor at three months following their initial appointment. Participants who received their final appointment with the welfare advisor fewer or later than three months following their initial appointment were contacted by their welfare advisor by telephone to complete their follow-up survey 3 months following their initial appointment. Non-respondents were contacted by their welfare advisors by telephone one week later as a prompt to complete the survey.

This follow-up period was chosen to increase the confidence in the association between advice receipt and changes to health and wellbeing, particularly in a multiply disadvantaged population, in which other factors could influence outcomes and underestimate the benefit of advice [27]. Secondly, it was chosen to minimise attrition, optimising statistical power and minimising the risk of bias [28, 29]. Finally, previous research has indicated a resolution time of three months for most cases [27].

### Outcome measures

#### Recruitment and retention

Recruitment and retention rates were calculated to establish the feasibility of recruiting and retaining participants for an evaluation of a welfare advice service co-located in a health setting within this ethnically diverse and deprived population. For this study, it was expected that there would be moderate recruitment rates, approximately 40–50% of all new referrals received, with low retention rates of approximately 20–30% for the follow-up survey.

#### Completeness of outcome measures

The acceptability and utility of the proposed evaluation tools to evaluate the impact of this intervention on the health, wellbeing and financial security of participants was assessed with respect to the completeness and missingness of the proposed outcome measures for these domains.

#### Participant sociodemographics

Sociodemographic data were routinely collected for participants by the VCS Alliance and were linked with survey responses. Responses available for each sociodemographic variable were detailed and high in number, in excess of ten responses available for each sociodemographic variable with the exception of gender and age. Therefore a number of sociodemographic variables were collapsed to facilitate analysis. Current relationship status was collapsed into four variables: ‘living with a partner’; ‘no longer living with partner’; ‘single’; and ‘widowed’. ‘Married’, ‘civil partnership’, and ‘co-habiting’ variables were collapsed into ‘living with partner’. ‘Divorced’, ‘formerly in a civil partnership’, and ’separated’ variables were collapsed into ‘no longer living with a partner’.

Ethnicity was coded using Census 2011 categories as ‘White British’, ‘Pakistani Heritage’ and ‘Other’. There were small numbers of non-White British and non-Pakistani Heritage mothers from multiple ethnic groups who were grouped and categorised within the ‘Other’ category.

Religion was coded as ‘Christian’, ‘Hindu’, Muslim and ‘Other’. There were small numbers of non-Christian, non-Hindu and non-Muslim participants who were grouped and categorised within the ‘Other’ category.

Preferred language was categorised as ‘English’, ‘Urdu’, “Punjabi’, ‘Mirpuri’ and ‘Other’. Similarly to the ethnicity and religion variables, there were small numbers of participants with a preferred language other than English, Urdu, Punjabi and Mirpuri, who were grouped and categorised within the ‘Other’ category.

Current health status was collapsed into five variables: ‘long-term health condition’; ‘physical or other disability’; ‘mental health condition’; ‘other’; and ‘none’. ‘Physical disability’, ‘visual impairment’, ‘learning disability’, and ‘hearing impairment’ were grouped and categorised as ‘physical or other disability’.

#### Self-reported financial security

To establish participant self-reported financial security, the surveys employed the question: ‘How well would you say you are managing financially right now?’ [30]. Answer options included: ‘living comfortably’; ‘doing alright’; ‘just about getting by’; ‘finding it quite difficult’; and ‘finding it very difficult’. The latter two options were grouped and categorised as indicating financial insecurity.

#### Mental health, wellbeing and health-related quality of life

Mental health, wellbeing and health-related quality of life were measured using the PHQ-8, SWEMWBS and EuroQol EQ-5D tools respectively [31–33]. Mental health was measured using the PHQ-8 questionnaire [31]. The scores from each item were summed to produce a total score between 0 and 24 points. Summed scores were used as a continuous variable with greater scores indicating a presence of depressive symptoms. Standard categorisations were employed for the scores: 0 to 4 no depression; 5 to 9 mild depression; 10 to 14 moderate depression; and 15 to 24 severe depression [34]. Symptoms suggestive of depression were defined as those with moderate to severe depression scores.

Wellbeing was measured using the seven-item SWEMWBS [35]. The score from each item was summed to produce a total score between 14 and 35. Summed scores were transformed and used as a continuous variable with greater scores indicating a more positive wellbeing. SWEMWBS scores were further categorised into low (7-19.5), average (19.6–27.4) and high (27.5–35) wellbeing groups.

The health-related quality of life of participants was measured using the five-item EQ-5D instrument (EQ-5D-5 L) [36]. These domains provide a descriptive profile that were transformed into health utility scores, based on UK societal preference weights for the health state, [37] ranging between 0 (representing death) and 1 (denoting perfect health). The EQ-5D-5 L questionnaire also includes a Visual Analog Scale, by which respondents can self-report their perceived health status with a continuous grade ranging from 0 (representing the worst possible health) to 100 (representing the best possible health).

All outcome measures were selected based on the experience of the Born in Bradford Research Programme and their successful use of these tools within the local community [38, 39]. Furthermore, the availability of a wide range of validated translated and transliterated versions of these tools was seen as important and necessary for an evaluation of a programme providing services to a diverse community with many preferred spoken languages.

### Financial outcomes

Data on the type of welfare advice provided and the financial outcome of individual client case work were routinely collected by the welfare advice providers and sent to the VCS Alliance monthly.

### Sample size

Loss to follow-up is a commonly identified challenge with the use of surveys and for evaluations of welfare advice services in particular [21]. A recently conducted evaluation of welfare advice services, using similar survey tools with a 3 month postal follow-up, achieved greater than 70% follow-up retention rates for both their advice and control groups. However, the use of financial incentives may explain the larger than average retention rates for this study [27]. The study recruited as many participants as possible in a twelve month period in order to increase the statistical power of the study to detect potential effect sizes.

The Difference ELicitation in TriAls (DELTA^2^) guidance provides specific guidance on choosing target differences in outcomes and on associated sample size calculations [40]. Sample size calculation is often based on a single primary outcome, the DELTA^2^ guidance advises that different candidate outcomes are considered in turn, and the corresponding sample size explored, particularly for complex interventions. Based on this guidance and given the lack of certainty regarding effect sizes for this intervention with respect to the chosen mental health, wellbeing and quality of life outcome measures, indicative sample sizes have been calculated and presented for each of the outcome measures respectively. This will improve the understanding of the potential effect size of this intervention and thus inform sample size calculations in future evaluations.

It was calculated that 1,151 participants would be required to have 90% power to detect a small effect on PHQ-8 scores, at 3 month follow-up compared to baseline, for a 5% two-sided alpha t-test. A sample size of 5,448 participants would be required to have 90% power to detect a small effect on EQ-5D-5 L health index scores, at 3 month follow-up compared to baseline, for a 5% two-sided alpha t-test. It was calculated that 505 participants would be required to have 90% power to detect a small effect on SWEMWBS scores, at 3 month follow-up compared to baseline, for a 5% two-sided alpha t-test.

### Data analysis

#### Self-reported financial security

Multiple logistic regression models were used to explore individual differences in self-reported financial security before and after the provision of welfare advice.

#### Mental health, wellbeing and health-related quality of life

Mean PHQ-8, transformed SWEMWBS, transformed EQ-5D-5 L utility scores and VAS scores are considered to approximate to a normal distribution with sufficient sample sizes [31, 41, 42]. Multiple linear regression models were used to explore individual change in PHQ-8, transformed SWEMWBS, transformed EQ-5D-5 L utility scores and VAS scores before and after the provision of welfare advice. To minimise regression to the mean, financial outcome data were also included in regression analyses where available. Where data were non-parametric Spearman’s rank co-efficient was used.

McNemar’s test was used to explore change in clinically relevant symptoms of depression, wellbeing and EQ-5D categories before and after the provision of welfare advice. Fischer’s exact test was used where sample sizes were small (< 5) for individual categorical variable stratum. Further details regarding presentation of data can be found in Table 1.

**Table 1:** Presentation of analysed data

Participant recruitment rate, retention rate and completeness of health, wellbeing and financial outcome measures are presented descriptively for participants. Baseline sociodemographics and all health, wellbeing and financial outcome measures are also presented descriptively for participants. Where data are parametric, mean values and standard deviation (SD) are presented.
Where data are non-parametric, median values and the interquartile range (IQR) are presented. Missing data on measures was small for most variables and was not adjusted for in the analyses. All statistical analyses were carried out using Stata 15 [55].

## Results

### Recruitment and retention

**Fig. 1:**
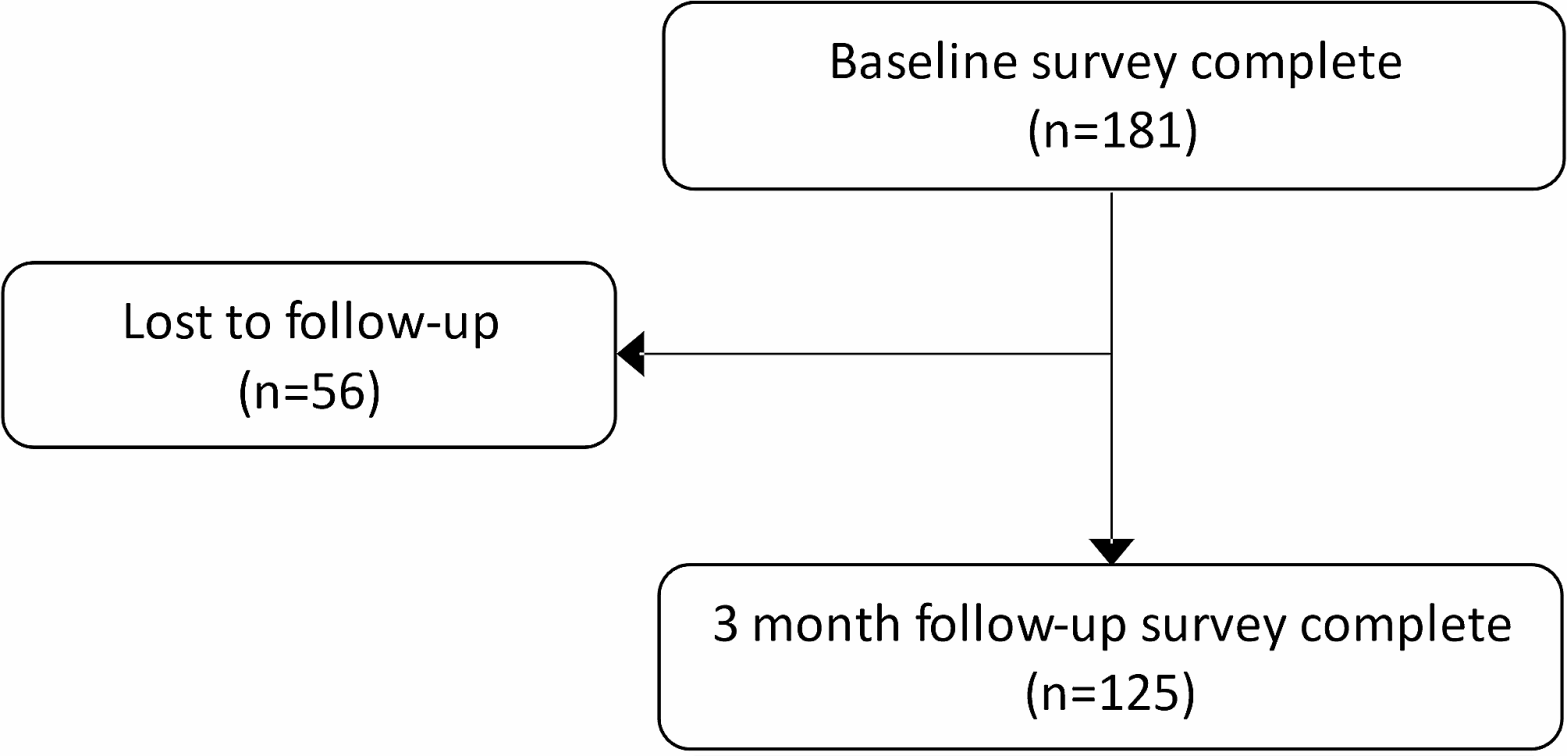
Consort diagram

Participants were recruited into the study between 1st March 2022 and 28th February 2023, with follow-up completed by 31st May 2023. During the study recruitment period, a total of 893 clients were referred into the VCS Alliance welfare advice programme. Of these clients, a total of 181 participants were recruited into the study, see Fig. 1. Thereby, the recruitment rate for this study was calculated as 20.3%. Of the 181 eligible participants recruited into the study, 125 participants completed the 3 month follow-up survey. The overall retention rate for this study was therefore calculated as 69%.

### Study population

Table 2 describes the sociodtemographics of the study participants who completed baseline and 3 month follow-up surveys (*n* = 125). Participants who were referred into the welfare advice service via the VCS Alliance welfare advice programme and consented to be part of the evaluation had a mean age of 49 (SD 11.8) years. A greater proportion of participants were female (63%) than male (36%). The majority of participants were living with a partner (59%), with 27% of participants being single, 10% being widowed and less than 5% reporting their current relationship status as no longer living with a partner. 33 (27%) participants reported that they were single parents.

**Table 2:** Sociodemographic characteristics of study participants, participants lost to follow-up and the general VCS Alliance population

	Study participants		Participants lost to follow-up		VCS Alliance population
	Number (*n* = 125)	Frequency (%) (95% CI)^*^	Number (*n* = 56)	Frequency (%) (95% CI)	Frequency (%) (95% CI)
Gender					
Female	63	63 (55–74)	26	47 (34–61)	62
Male	36	36 (26–74)	29	53 (39–66)	38
Missing	26		< 5		
Age					
18–24	< 5	< 5	< 5	< 5	7
25–34	6	6 (3–13)	< 5	< 5	13
35–44	34	34 (26–44)	< 5	< 5	24
45–54	32	32 (24–42)	13	23 (14–36)	24
55–64	12	12 (7–20)	37	66 (53–77)	17
65 and above	15	15 (9–24)	< 5	< 5	15
Missing	24		< 5		
Current relationship status					
Living with partner	59	60 (50–69)	37	68 (55–80)	54
No longer living with partner	< 5	< 5	< 5	2 (0–13)	2
Single	27	27 (19–37)	10	19 (10–31)	38
Widowed	10	10 (5–18)	6	11 (5–23)	6
Missing	25		< 5		
Whether single parent					
Yes	33	27 (20–35)	19	35 (23–48)	-
No	91	73 (65–80)	36	65 (52–77)	-
Missing	< 5		< 5		
Ethnicity					
Pakistani Heritage	82	87 (81–96)	20	36 (29–41)	69
White British	< 5	< 5	31	55 (46–60)	9
Other	10	9 (5–14)	< 5	< 5	22
Missing	30		< 5		
Religion					
Christian	< 5	< 5	17	38 (25–53)	12
Hindu	< 5	< 5	< 5	< 5	1
Muslim	89	94 (86–97)	25	56 (41–70)	73
Other	< 5	< 5	< 5	< 5	14
Missing	23		11		
Preferred language					
English	15	15 (9–24)	46	84 (71–91)	58
Urdu	18	18 (12–27)	< 5	< 5	25
Punjabi	29	30 (21–39)	7	13 (6–25)	6
Mirpuri	18	18 (12–27)	< 5	< 5	1
Other	18	18 (12–27)	< 5	< 5	10
Missing	27		< 5		
Self-reported health issues					
Long term health condition	9	9 (5–17)	21	38 (27–53)	12
Physical or other disability	29	30 (21–39)	< 5	2 (0–6)	32
Mental health condition	31	32 (23–42)	9	17 (9–29)	21
Other	< 5	< 5	15	27 (17–41)	9
None	25	26 (18–35)	9	16 (9–29)	26
Missing	27		< 5		
Employment status of main earner in household					
Employed	15	15 (9–23)	14	26 (16–39)	-
Self-employed	7	7 (3–14)	< 5	6 (2–16)	-
Unemployed	81	79 (70–86)	36	67 (53–78)	-
Missing	22		< 5		
Worry about job security of main earner in household					
Yes	20	16 (10–24)	21	39 (27–53)	-
No	7	6 (3–24)	6	11 (5–23)	-
Don’t know	97	78 (70–85)	27	50 (37–63)	-
Missing	< 5		< 5		
Worry about eviction					
Never	29	23 (17–32)	20	35 (24–49)	-
Sometimes	31	25 (18–33)	25	45 (32–58)	-
Often	64	52 (43–60)	11	20 (11–32)	-
Missing	< 5		< 5		
Worry about whether food will last					
Never	19	15 (10–23)	23	41	-
Sometimes	41	33 (25–42)	21	37	-
Often	65	52 (43–61)	12	22	-
Missing	10		< 5		

^*^Frequency calculations do not include missing data

- denotes data that is unavailable

Participants were predominately of Pakistani Heritage: 82 (87%) were of Pakistani Heritage; less than 5% identified as White British; and 10 (9%) were of other ethnic groups. There was a wide range of preferred languages reported by participants: 29 (30%) reported their preferred language as Punjabi; 18 (18%) as Mirpuri; 18 (18%) as Urdu; 15 (15%) as English; and 18 (18%) as another language.

Over half of participants (58%) reported that they had a physical or mental health concern at the time of accessing the welfare advice service: 31 (32%) reported having a mental health condition; 29 (30%) reported a physical or other disability; and 9 (9%) reported a long term health condition.

The majority of participants reported that the main earner in the household was unemployed (79%). A small proportion of participants reported that the main earner in the household was employed (15%) or self-employed (7%). 16% of participants were worried about the job security of the main earner in the household over the next year, compared to 6% of participants who were not.

At the time of accessing welfare advice services, most participants (64%) reported that they often worried about eviction or losing their home, compared to 25% of participants who sometimes worried about losing their home and 23% of participants who never worried. Similarly, most participants (52%) reported that they often worried about food lasting, compared to 33% of participants who sometimes worried about losing their home and 15% of participants who never worried.

There were some differences observed between the study population and the general VCS Alliance population. The majority of the study population were between the ages of 35–44 (34%) and 45–54 (32%), whereas there was a smaller proportion of people in these age groups in the general population (24% and 24% respectively), with greater numbers and more even spread of people across other age groups. There was a greater proportion of Pakistani Heritage participants in the study population (87%) compared to the general VCS Alliance population (69%), with a greater proportion of study participants in the ‘Other’ (9%) ethnic group compared to the general study population (22%). Participants in the study population were more likely to be of Muslim faith (94%) and less likely to be of Christian faith (< 5%) compared to the general population (73% and 12% respectively). Study participants were less likely to report English as their first language (15%) compared to the general population (58%) and more likely to report Punjabi (30%) and Mirpuri (18%) as their preferred language compared to the general population (6% and 1% respectively).

Some differences were observed between participants who engaged in follow-up and participants lost to follow-up. Participants who were lost to follow-up were slightly older with a mean age of 56 (SD 9.5) years. There was a greater proportion of males in the lost to follow-up group (53%) compared to the general study participants (36%), creating a more balanced split between males and females. Participants who were lost to follow-up were more likely to be White British (55%) and report English as their preferred language (84%) compared to those who participated in the follow-up (less than 5% and 15% respectively). More participants who were lost to follow-up lived in a household where the main earner was employed (26%) compared to those followed up at 3 months (15%) and fewer participants lived in a household where the main earner was unemployed (67%) compared to the follow-up group (79%). A greater proportion of participants who were lost to follow-up reported having a long term health condition (38%) and were less likely to have a physical disability (2%) or mental health condition (17%) than study participants who were not lost to follow-up (9%, 30% and 17% respectively). Finally, participants who were lost to follow-up were less likely to report being worried about eviction (35%) and whether food will last (41%), reporting that they never worried about these issues, compared to those not lost to follow-up (23% and 15% respectively). There was no observed difference in current relationship status, single parent status or employment status between those who participated in the 3 month follow-up and those who did not.

### Completeness of outcome measures

The completeness of outcome measures was calculated at baseline, 3 month follow-up and overall for the study evaluation with respect to: self-reported financial security; mental health, wellbeing and health-related quality of life; and financial outcome measures, see Table 3.

**Table 3:** Completeness of outcome measures at baseline, 3 month follow-up and overall for the study evaluation

	Baseline (*n* = 181)		Follow-up (*n* = 125)		
Outcome measure	Number (n)	Completeness (%)	Number (n)	Completeness (%)	Overall (%)
**SOCIOECONOMIC SECURITY**					
Worry about job security of main earner in household	177	98%	-	-	98%
Worry about eviction	180	99%	-	-	99%
Worry about whether food will last	170	94%	-	-	94%
Self-reported financial security	180	99%	119	95%	98%
**HEALTH, WELLBEING AND HEALTH-RELATED QUALITY OF LIFE**					
PHQ-8 Score	180	99%	125	100%	99%
SWEMWBS Score	180	99%	124	99%	99%
EQ-5D VAS score	180	99%	124	99%	99%
EQ-5D health state index score	180	99%	124	99%	99%
**FINANCIAL OUTCOMES**					
Type of welfare advice case work	-	-	125	100%	100%
Financial gains	-	-	56	45%	45%

Overall, the majority of key outcome measures were extremely well completed. The additional sociodemographic variables added to the baseline survey questionnaire to improve understanding of participant socioeconomic security and how this is experienced were completed by most participants (94–99%). Participant response rate for self-reported financial security was also high at follow-up (95%). The completeness of health, wellbeing and health-related quality of life outcomes was universally high (99%) across the evaluation overall.

Completeness of financial outcome measures collected by the VCS Alliance providers was variable. The completeness of type of welfare advice case work managed for participants was high (100%). However, it was difficult to know whether any types of case work were missing given that more than one type of case work was often managed per participant. Financial outcomes were not well completed in comparison to other outcome measures (45%). The majority of participants (55%) were documented to be still awaiting the outcome of their claims. No detail was included on any debt managed.

### Self-reported financial security

Most participants reported that they were finding it very (50%) or quite (15%) difficult to get by financially or were just about getting by (25%). Few participants reported living comfortably (less than 5%) or doing alright (7%) at the time of accessing the welfare advice services.

Fewer participants reported feeling financially insecure at their 3 month follow-up appointment (59.42% 95% CI 50.68%, 68.11%) compared to baseline (64.31% 95% CI 56.53%, 73.68%), see Table 4. The difference between these groups was small and the reported *p*-value for this difference was 0.059 suggesting that there may be little evidence for the utility of this outcome measure to detect a change at this point in time.

**Table 4:** Effects of intervention on participant health, wellbeing and financial security

	Baseline		3 month follow-up		
	Number	Frequency (%) (95% CI)	Number	Frequency (%) (95% CI)	*p*-value
**FINANCIAL SECURITY**					
**Self-reported financial insecurity (Baseline n = 125, Follow-up n = 119)**					
Secure	44	35.59 (27.32–44.47)	48	40.58 (32.89–49.32)	0.059
Insecure	81	64.41 (56.53–73.68)	71	59.42 (50.68–68.11)	0.059
**MENTAL HEALTH**					
**PHQ total score**	**Median**	**Interquartile range**	**Median**	**Interquartile range**	
PHQ total score	13.00	4.00–20.00	12.00	2.50–19.50	0.344
**Clinically relevant symptoms of depression**	**Number**	**Frequency (%) (95% CI)**	**Number**	**Frequency (%) (95% CI)**	
No or few clinically relevant symptoms of depression	51	40.80 (32.31–49.17)	54	43.31 (34.10-52.09)	0.414
Clinically relevant symptoms of depression	74	59.20 (50.83–67.69)	71	56.69 (47.91–65.90)	0.414
**WELLBEING**					
**SWEMWBS score**	**Median**	**Interquartile range**	**Median**	**Interquartile range**	
Adjusted score	17.98	15.32–23.35	19.25	15.84–24.11	0.048
**SWEMWBS category**	**Number**	**Frequency (%) (95% CI)**	**Number**	**Frequency (%) (95% CI)**	
High wellbeing	76	60.73 (51.96-69.00)	73	58.54 (49.76–67.47)	0.027
Average wellbeing	26	20.08 (14.90-28.25)	33	26.04 (19.72–35.28)	0.027
Low wellbeing	23	23.19 (12.49–26.11)	18	14.42 (9.83–22.67)	0.027
**HEALTH-RELATED QUALITY OF LIFE**					
**EQ-5D-5 L score**	**Mean**	**Standard deviation**	**Mean**	**Standard deviation**	
VAS score	50.82	25.40	54.93	27.35	< 0.001
**EQ-5D health state index score**	**Number**	**Interquartile range**	**Number**	**Interquartile range**	
EQ-5D health state index score	0.4535	0.117–0.887	0.587	0.100-0.887	< 0.001

### Mental health, wellbeing and health-related quality of life

Following access to welfare advice services, participants experienced improvements across all mental health, wellbeing and health-related quality of life domains. Mean group PHQ-8 scores fell from 13.00 (IQR 4.00, 20.00) at baseline to 12.00 (IQR 2.50, 19.50) at 3 month follow-up. The proportion of participants with symptoms suggestive of clinical depression fell from 59.20% (95% CI 50.83%, 67.69%) to 56.69% (95% CI 47.91%, 65.90%). This change was small and not statistically significant (*p* = 0.344 and 0.414 respectively). However, the sample size was not sufficient to detect any meaningful change should there be one.

Wellbeing improved between baseline and follow-up following receipt of welfare advice and support. Mean group adjusted SWEMWBS scores improved from 17.98 (IQR 15.32, 23.35) at baseline appointments to 19.25 (IQR 15.84–24.11) at follow-up appointments. Following access to services, a greater proportion of participants were found to have average wellbeing categorical scores (26.04% 95% CI 19.72%, 35.28%) compared to baseline (20.08% 95% CI 14.90%, 28.25%) and a smaller proportion of participants were found to have low wellbeing categorical scores (14.42% 95% CI 9.83%, 22.67%) compared to baseline (23.19% 95% CI 12.49%, 26.11%). Improvements in mean group participant wellbeing SWEMWBS scores and improved wellbeing categories were found to be statistically significant, demonstrating evidence of promise for improvements in wellbeing following access to services (*p* = 0.048 and 0.027 respectively).

Mean group EQ-5D-5 L VAS scores improved from 50.82 (SD 25.40) at baseline to 54.93 (SD 27.35) following welfare advice at 3 month follow-up. Mean group EQ-5D health state index scores also demonstrated improvements from 0.4535 (IQR 0.117, 0.887) at baseline to 0.587 (IQR 0.100, 0.887) at 3 month follow-up. Improvements in mean group participant health-related quality of life VAS scores and health state index scores were found to be statistically significant, demonstrating evidence of promise for improvements in wellbeing following access to services (*p* < 0.001 respectively).

### Financial outcomes

The VCS Alliance welfare advice programme provided a wide range of welfare advice and support to participants throughout the evaluation period. Participants often received advice and support on more than one issue. There were a total of 220 welfare advice issues managed by the welfare advisors with an average of 1.76 welfare advice work issues managed per participant. The most frequent welfare advice and support provided was associated with benefits eligibility checks and applications (5%), including Personal Independence Payments (23%), Universal Credit (11%), Disability Living Allowance (5%), Attendance Allowance (3%), Working Tax Credit (3%) and Carers Allowance (3%). Welfare advisors also commonly provided support with utility bills (5%), council tax (5%) and housing issues (5%).

Of the 125 participants who completed follow-up, 56 participants had complete financial outcome data. For these participants, the welfare advice service generated a total financial gains of £21,823.05. Participants with complete data on their financial outcome gained an average of £389.70 per participant with a range of £0 to £9,878.45 awarded per participant following access to the service.

## Discussion

### Summary of key findings

This research describes some of the key impacts of a welfare advice service co-located in primary care on participant health, wellbeing and financial security. It explores whether the proposed evaluation tools are suitable to evaluate this intervention with respect to recruitment rate, retention rate and completeness of outcome measures.

Overall, there were low participant recruitment rates into the study. It is unclear to what degree this reflects a lack of engagement from some of the welfare providers and associated welfare advisors and a lack of engagement from potential participants. The calculated retention rate (69%) was high and comparable to similarly conducted evaluations [27]. Explanations for this could include the timing of follow-up to fall in line with completion of welfare advice case work and the use of financial incentives, which is also comparable to similarly conducted evaluations [27].

There were some significant differences between the study population and participants lost to follow-up. Notably, participants who were lost to follow-up were more likely to be White British (55%) and report English as their preferred language (84%) compared to those who participated in follow-up (less than 5% and 15% respectively). More participants who were lost to follow-up lived in a household where the main earner was employed (26%) compared to those followed up at 3 months (15%) and fewer participants lived in a household where the main earner was unemployed (67%) compared to the follow-up group (79%). Moreover, participants who were lost to follow-up were less likely to report being worried about eviction (35%) and whether food will last (41%), reporting that they never worried about these issues, compared to those not lost to follow-up (23% and 15% respectively). These findings could suggest that participants who were lost to follow-up were reflective of the more financially secure participants.

Overall, the majority of key outcome measures were well completed, indicating participant acceptability of these measures in this population. Participant response rate for job security of the main earner in the household was also high (98%), however the majority of participants reported that they did not know whether they were concerned about the employment status of the main earner in the household (78%). This uncertainty may reflect the high unemployment rate of the main earners in the household of this participant group (79%) who may have not found this question relevant or may have found this question difficult to answer for this reason. Financial gains outcomes were not well completed in comparison to other outcome measures (45%).

There was evidence suggestive of an improvement in the felt and lived experience of financial security for participants following access to these services. However, this improvement was small and demonstrated little evidence of promise of a significant impact on self-reported financial security following access to services. A longer duration of follow-up may be required to detect a difference in financial security following access to services.

This evaluation also demonstrated evidence of promise for improvements in measured wellbeing and health-related quality of life for participants accessing services in a highly ethnically diverse population. There were small improvements in group mental health, as indicated by PHQ-8 scores, however this study was not powered sufficiently to detect any meaningful change in PHQ-8 scores. Given that no control group was included for this study, it is not clear whether these associations are causal and the role of chance cannot be excluded.

This is the first known evaluation to utilise PHQ-8 and EQ-5D instruments to measure mental health and health-related quality of life in an evaluation of welfare advice services co-located in a health setting. Woodhead et al. reported improved wellbeing scores for participants whose advice resulted in positive outcomes (ß 1.29, 95% CI 0.25–2.32) [27]. Krska et al. also reported preliminary findings of improved WEMWBS scores at 3 month follow-up following receipt of welfare advice within a primary care setting, although these improvements were not quantified [43]. A study published by Howel et al. exploring the financial and health-related quality of life impacts of a co-located welfare advice service in a similarly deprived population found no intervention effect. Howel et al. explored outcomes at 24 months and suggests that where improvements might exist, they may not persist beyond this time [23].

Overall, the VCS Alliance welfare advice programme generated a total of £21,823.05 for all participants, with participants gaining an average of £389.70 per participant for participants with complete financial outcome data. Financial outcomes for participants of this study are lower in value in comparison to other published studies. Participants from the studies included in the recently published systematic review of welfare advice services co-located in health settings gained on average £1,840, with a range of £776 to £3,656 gained on average per participant between published studies [22, 27, 44–48]. Similarly, as previously reported, financial outcome measures for this evaluation were not well completed. Therefore, it is possible that the financial outcomes are likely to be significantly under-estimated.

### Limitations

There is a lack of understanding regarding the reasons for the low rates of recruitment and retention at follow-up. Given the inclusion of nine discrete welfare advice services, there is the potential for a significant degree of inter and intra-service variability that could contribute to these findings, in addition to participant related factors. This low recruitment rate overall introduces the possibility of selection bias and therefore misleading findings. Furthermore, satisfaction with the service provided may affect the likelihood of participant engagement with follow-up, leading to potential overestimation of measured associations between welfare advice services and health and wellbeing outcomes. In order to reduce administrative burden and improve recruitment and retention rates, welfare advisors were also used to administrate participant questionnaires. This approach may have introduced response bias, with participants being inclined to provide positive responses at follow-up. Comparing results with other studies of similar and differing populations is important to gain a fuller picture of the impact of co-located welfare advice services on mental health, wellbeing and health-related quality of life.

Variation in reported outcome measures was not examined with respect to temporality, where there might have been seasonal themes emergent throughout the course of the year. Such variation could be expected during colder months where families face greater household costs with increased need for energy coupled with rising energy costs. Similarly, the analysis does not take into account additional temporary or alternative sources of financial support received by families, for example the Energy Bills Discount Scheme, Warm House Discounts and Cost of Living Payments introduced by the UK Government in 2023 [49]. Furthermore, there is increasing prevalence of alternative and non-documented sources of financial support being used by families. Informal rotating savings and credit associations (ROSCA), often referred to as committees, have been formed within Pakistani Heritage communities in Bradford as a means to establishing financial resilience for families [50]. Such undocumented forms of financial support have the potential to introduce confounding into the results.

In order to minimise regression to the mean, financial outcome data were included in the regression analyses. However, regression to the mean may persist despite these measures given that financial outcome data was not well completed. Three months was chosen as an appropriate follow-up time period given its feasibility to implement within the context of this service, with respect to average case completion time, and in order to maximise retention. However, it is still not clear what time period is sufficient to measure changes to health and wellbeing outcomes attributable to access to a welfare advice service. Shorter time frames may not allow the full effects to be measured and longer follow-up periods may allow for further regression to the mean.

### Implication of findings

This is the first evaluation of welfare advice services co-located in health settings to formally consider and offer evidence of feasibility for the recruitment and retention of participants for an evaluation of a welfare advice service co-located in a health setting within an ethnically diverse and deprived population. Future research in this area should give consideration to the method of recruitment of potential participants to ensure selection bias is minimised. Recruitment should be conducted by an independent researcher and recruitment offered to all potential participants in a standardised manner to minimise selection bias. Where possible, follow-up data collection should also be performed by an independent researcher to minimise observation bias.

This study offers evidence of acceptability and utility of the proposed evaluation tools to evaluate the impact of this intervention on the health, wellbeing and financial security of participants with respect to completeness of outcome measures and their ability to detect potential change in outcome measures for the intervention in this population. Chosen measures of mental health, wellbeing and health-related quality of life were extremely well completed and can be considered acceptable for use in the evaluation of this intervention in this setting and within this unique and diverse population. It is clear that effort is required on the part of the research and administrative teams to follow-up financial outcome data in order to facilitate an appropriate economic analysis. Other approaches to obtaining timely, accurate and validated outcome measures should be considered to facilitate evaluations of co-located welfare advice services, for example routine data linkage.

This study also offers evidence of promise that welfare advice services co-located in health settings improve wellbeing and health-related quality of life in a highly ethnically diverse population, living in the most deprived centiles in the UK. There was little evidence to suggest that this intervention improves mental health, by means of improved PHQ-8 scores, however this study was not powered to detect small effect sizes with respect to change in PHQ-8 scores. Overall, the potential of the outcome measures utilised for this evaluation to detect potential changes in mental health, wellbeing and health-related quality of life is positive even at short follow-up intervals. These indicative effect sizes can be utilised to guide sample sizes calculations of future evaluations. Future research could also consider the use of an additional follow-up period at six months to assess how impact to financial, mental health, wellbeing and health-related quality of life outcomes change over time.

Inconsistencies in measured outcomes makes synthesis of evidence difficult, therefore the use of core outcome sets could be considered for future research and development in this area. The use of a core outcomes set has been promoted to harmonise the outcomes used, to facilitate meta-analysis where appropriate, particularly where achieving sufficient sample sizes may be challenging, and to ensure that key stakeholders are consulted on the relevance of what is being measured in evaluations [51, 52]. The Core Outcome Measures in Effectiveness Trials (COMET) initiative supports the development of core outcome sets, largely for clinical trials, although it includes some resources that may be more widely applicable [53]. However, a core outcome set is not yet available for welfare advice services. Indeed, few core outcome sets have been adapted specifically for public health research in the UK. A core outcome set for early years (COS-EY) has recently been published to increase standardisation and guide the selection of outcome measures for systems-based evaluation of public health programmes and supports evaluation of individual interventions within system change approaches [54]. Whilst this may prove useful for this complex intervention, before this core outcome set can be fully implemented, the authors highlight that further work is undertaken to confirm the definition of each outcome, prior to deciding on the most appropriate measures or data sources [54].

## Conclusion

With an ever increasing cost of living, energy prices and inflation in the midst of the recovery of the COVID-19 pandemic, the ability of people to improve their financial security is untenable without intervention. The need for policy makers and commissioners to act to support vulnerable people is now urgent and critical to prevent further financial, fuel and food debt, homelessness, poor health and widening existing health and social inequalities. It is important that high quality and well evaluated interventions are implemented to achieve this.

Existing published literature evaluating the impact of welfare advice services co-located in health settings has published evidence suggestive of improvements to financial security and to the health and wellbeing of participants in receipt of welfare advice co-located in a health setting, leading to potential reductions in health inequalities. However, no consensus has been achieved on the most appropriate measures for these outcomes, nor an appropriate time frame within which to follow-up participants, particularly in a diverse and deprived population.

This research demonstrates the feasibility of evaluating a welfare advice service co-located in primary care in a deprived and ethnically diverse setting, utilising the PHQ-8, SWEMWBS and EuroQol EQ-5D tools, as measures of mental health, wellbeing and health-related quality of life respectively. This research highlights the importance of achieving adequate completeness of financial outcome measures, with respect to financial outcomes for participants. These outcomes measures are important to fully understand the impact of the services on participant financial security and how this interplays with other factors, such as participant health and wellbeing. Finally, this research provides further evidence of promise to support the hypothesis that the implementation of a welfare advice service co-located in a health setting can improve health and wellbeing and reduce health inequalities.

## Data Availability

This data is available through a system of managed Open Access. Researchers who would like access to this data, or any other Born in Bradford data are encouraged to submit an expression of interest which will be reviewed by the BiB Executive (who meet to review proposals on a monthly basis and will endeavour to respond to your request as soon as possible). If your request is approved we will ask you to sign a Data Sharing Contract and a Data Sharing Agreement. For further information please see: How to access data - Born In Bradford. Alternatively, please contact sianreece@doctors.org.uk for further information.

## Abbreviations

COMET: Core Outcome Measures in Effectiveness Trials
COS-EY: Core Outcome Set for Early Years
IQR: Interquartile range
NIHR: National Institute for Health and Care Research
OR: Odds ratio
PHQ-8: Patient Health Questionnaire
SD: Standard deviation
SWEMWBS: Shortened Warwick-Edinburgh Mental Wellbeing Scale
VAS: Visual Analogue Scale
VCS: Voluntary and Community Sector
WEMWBS: Warwick-Edinburgh Mental Wellbeing Scale
